# Building Responsive Health Systems to Help Communities Affected by Migration: An International Delphi Consensus

**DOI:** 10.3390/ijerph14020144

**Published:** 2017-02-03

**Authors:** Kevin Pottie, Charles Hui, Prinon Rahman, David Ingleby, Elie A. Akl, Grant Russell, Li Ling, Kolitha Wickramage, Davide Mosca, Claire D. Brindis

**Affiliations:** 1 Departments of Family Medicine & Epidemiology and Community Medicine, University of Ottawa, 1 Stewart Street, Ottawa, ON K1N 6N5, Canada; 2 Bruyère Research Institute, 85 Primrose Ave, Annex E-208, Ottawa, ON K1R 6M1, Canada; Prinon.Rahman@dal.ca; 3 Department of Pediatrics, University of Ottawa, Ottawa, ON K1N 6N5, Canada; chui@cheo.on.ca; 4 Centre for Social Science and Global Health, University of Amsterdam, Nieuwe Achtergracht 166, 1018 WV Amsterdam, The Netherlands; j.d.Ingleby@uu.nl; 5 Department of Internal Medicine, Faculty of Medicine, American University of Beirut, Beirut 11-0236, Lebanon; ea32@aub.edu.lb; 6 School of Primary Health Care, Monash University, Notting Hill Campus, Bldg 1, 270 Ferntree Gully Road, Notting Hill VIC 3168, Australia; grant.russell@monash.edu; 7 Center for Migrant Health Policy, San Yat-sen University School of Public Health, No.74 Zhongshan Er Road, Guangzhou 510000, China; lingli@mail.sysu.edu.cn; 8 Migration Health Division, International Organization for Migration, 17, Route des Morillons, CH-1211 Geneva, Switzerland; kwickramage@iom.int (K.W.); dmosca@iom.int (D.M.); 9 Philip R. Lee Institute for Health Policy Studies, University of California, San Francisco, 3333 California Street, Suite 265, San Francisco, CA 94118, USA; claire.brindis@ucsf.edu

**Keywords:** public health, health systems, refugees, other migrants, Delphi consensus

## Abstract

Persons affected by migration require health systems that are responsive and adaptable to the needs of both disadvantaged migrants and non-migrant populations. The objective of this study is to support health systems for populations affected by migration. Materials and Methods: An international Delphi consensus process was used to identify policy approaches to improve health systems for populations affected by migration. Participants were leading migrant health experts from Americas, Europe, Middle East, Asia, and Australasia. We calculated average ranking scores and qualitatively analyzed open-ended questions. Results: Participants identified the following key areas as priorities for policy development: health inequities, system discrimination, migrant related health data, disadvantaged migrant sub-groups, and considerations for disadvantaged non-migrant populations. Highly ranked items to improve health systems were: Health Equity Impact Assessment, evidence based guidelines, and the International Organization for Migration annual reports. Discussion: Policy makers need tools, data and resources to address health systems challenges. Policies need to avoid preventable deaths of migrants and barriers to basic health services.

## 1. Introduction

In 2015, more than 1.2 million asylum seekers and other migrants arrived in Europe in a short period of time and thousands more died in transit. The “European migrant crisis” led to a call for humanitarian action and a World Health Organization (WHO) call for responsive health systems around the world. The WHO responsiveness framework’s aims help populations equitably meet core social goals and international human rights. Populations also expect the health system to treat people with dignity. Within this framework, health system responsiveness was given the formal definition of “the ability of the health system to meet the population’s legitimate expectations regarding their interaction with the health system, apart from expectations for improvements in health or wealth” (WHO Health System Responsiveness http://www.who.int/responsiveness/en/). Modern populations are rarely homogenous, and the International Organization for Migration (IOM) reports global international migration is expected to double from 232 million to more than 400 million by 2050 [1]. This paper identified and prioritized policy challenges to support responsive health systems for the growing internal and international migration movements. 

Refugees, asylum seekers and other migrants (e.g., skilled/economic/family migrant) may unintentionally cause unexpected stress to local health systems. Health systems may require interpreter services and additional service capacity, and migrants may need special health coverage [2,3]. Migrant patients may need care for diseases unfamiliar to the local health system such as malnutrition, tuberculosis, HIV, Hepatitis C, and intestinal parasites, or add burdens on health systems through added demand from dental disease and post-traumatic stress disorder (PTSD) [4]. Finally, policy makers need to be aware of the effect that migration can have on local disadvantaged populations when the system is not prepared for migration. The example of the Lebanese migration crisis demonstrated that in refugee crisis scenarios, the local disadvantaged population may suffer from limited access to food, affordable shelter and basic healthcare. In Europe and North America, migration may place pressure on local disadvantaged populations dependent on public social services if authorities do not take timely measures to respond to increased demand [5]. 

Traditionally, most political jurisdictions have not considered mass migration in their health system planning [6,7]. In some countries, health systems struggle to accommodate migrants and rarely are they considered in health system planning [7]. Certain countries, such as Australia, Canada, the United States, have adopted explicit migrant health policies [8,9]. Evidence based approaches may help develop consensus and marshal political will, strengthen policy-making capacity and service delivery [10]. 

Decision makers are often faced with competing challenges, limited knowledge and limited financial resources. Efforts to prioritize policy challenges and the transfer of knowledge can improve health systems [11]. The objective of this study was to prioritize needs and challenges for building responsive health systems for refugees, other migrants and groups affected by migration. This Delphi consensus aimed to identifying the: (1) populations affected by migration; (2) challenges in building a responsive health system; and (3) policy development and health systems implementation challenges for refugees, other migrants.

## 2. Materials and Methods

A scoping literature review [7] and an international workshop at the Institute of Population Health, Ottawa (2014), identified a need for migrant health data, local migrant partnerships, and resources for decision makers for Health Equity Impact Assessment (HEIA) frameworks [12]. 

### 2.1. Study Design

We conducted a Delphi consensus process to set priorities for building responsive health systems for refugees, other migrants and disadvantaged persons affected by migration. For the purposes of this paper, local disadvantaged populations are defined by low-socioeconomic status, disability and/or advanced age. This online consensus process involved a series of three phases conducted between 16 December 2015 and 24 May 2016. All three phases used Survey Monkey software (www.surveymonkey.net). Each subsequent phase provided information to the participants regarding the stage of the Delphi consensus, emerging results and the priority setting criteria. This study received ethics approval from The Ottawa Health Science Network Research Ethics Board (OHSN-REB) and Bruyère Research Ethics Board (#M16-15-031). 

### 2.2. Sample/Participants 

We invited leading migrant health experts from Australia, Asia, Europe, Middle East, North America and South America. These experts nominated additional stakeholders from their region.

The additional participants came from Canada, China, Denmark, Germany, Greece, Italy, Lebanon, Malaysia, The Netherlands, Spain, Sweden, and U.S.—all countries that have ongoing migration pressures. Experts selected stakeholders from the following groups to ensure a broad range of expertise and experience: primary health care practitioners; public health and/or surveillance professionals; policy developers; health researchers; community health workers; and health impact assessment developers. 

### 2.3. Delphi Surveys

**Phase 1**: Stakeholders were asked to: define disadvantaged persons affected by migration; and suggest other nomenclature on local disadvantaged populations affected by migration from their country perspective. We also asked experts to highlight any policy and practice based tools or resources they were aware of, and/or used, from a list of 9 pre-identified tools/resources and to identify other tools that were used. The experts were also asked “to rank specific health system challenges”. 

**Phase 2**: First, we shared with the stakeholders a summary of responses to the first survey. We then asked the following health system questions: who are the migrant population of interest? What other local disadvantaged populations may be affected by migration? 

We then asked the stakeholders about challenges associated with: migrant-relevant health policies, migrant specific data, engaging migrant communities, and implementation of policies. The panel was then asked to identify additional tools/methods to help address these challenges.

**Phase 3**: We shared the results of previous surveys and presented the highest ranked challenges for responsive health system policy planning. We asked stakeholders to comment on this ranking and to offer additional insights. 

### 2.4. Data Analysis 

We analyzed stakeholder characteristics and then the panel’s responses, calculating group percentages and average ranking scores for rank questions. For each phase, we compared demographic characteristics of stakeholders who did not respond to that phase with respondents who did. Qualitative analysis was done for open-ended questions. We descriptively analyzed the qualitative responses in a table and identified key themes based on repetition of concept words. Outliers were gathered, discussed in subsequent surveys and reported when unresolved. In addition, we performed rank-shift analyses on key planning challenges to evaluate the shift on agreement between the second and final phase. 

## 3. Results

**Figure 1:**
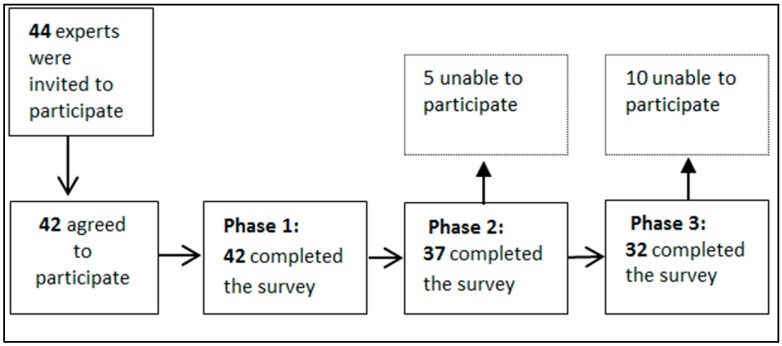
Delphi expert response rates over three phases.

**Phase 1**: Out of the 44 stakeholders we invited to participate, 42 (95%) responded (see Figure 1). Table 1 provides the characteristics of the respondents. The stakeholders came from Oceana, Europe, Asia and North America; 47.2% of them reported that English was not their first language. Most stakeholders (75%) were migration health researchers, public health and/or surveillance professionals or primary healthcare practitioners. Fifty-two percent had more than 10 years of experience in the field of migrant health.

**Figure 2:**
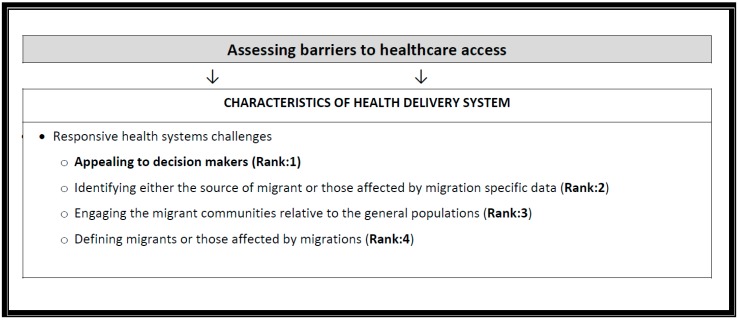
Priority ranked results on health system challenges and characteristics of disadvantaged populations.

In this phase, experts ranked priorities associated with building a responsive and inclusive health system (see Figure 2). 

### 3.1. Disadvantaged Persons Affected by Migration 

Stakeholders described disadvantaged persons affected by migration were “forced migrants, asylum seekers”, “trafficked women”, “discrimination with respect to access to employment, low wages, and additional barriers for immigrant women”, and “receiving country communities”. From the experts’ text responses, three themes were identified in describing disadvantaged persons affected by migration as follows: (1) belonging to a subgroup of migrants at risk for health inequities; (2) those subjected to political and system exclusions; (3) local members of the receiving communities facing competition due to mass migrations; and (4) Intersectionality (intersectionality considers simultaneous interactions among race, gender and class and other social locations and identities (e.g., immigration status, sexuality, literacy and religion)) *between immigrant status, ethnicity and inequity*, which was reported by only one expert [13]. 

Summaries of responses describing disadvantaged populations, awareness and use of practice-based tools and resources are provided in Appendix Table A1, Table A2 and Table A3.

### 3.2. Awareness and Use of Practical Resources 

Out of the nine resources listed, the majority of experts were aware of (86%) and reported using (59%) the International Organization for Migration Annual Reports and other supporting publications (see Appendix Table A2 and Table A3). Figure 2 illustrates the ranked challenges associated with building a responsive and inclusive health system. Stakeholders ranked “appealing to decision makers” as the most important policy priority to address.

**Figure 3:**
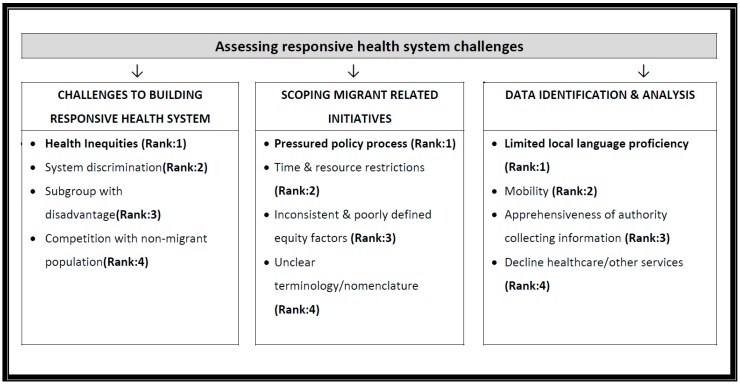
Priority ranked results on assessing responsive health system challenges.

**Figure 4:**
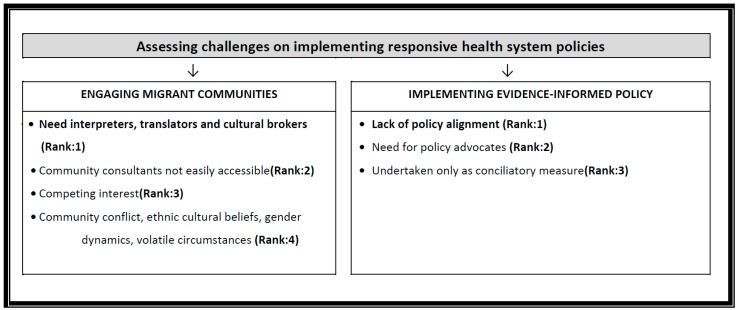
Priority ranked results on responsive health system implementation challenges.

**Phase 2**: The results for the second phase identified key challenges to developing and implementing Responsive health system policies. The response rate for this phase was 83%. The top ranked priority challenges for developing responsive health system policies were: health inequities, the pressured policy process in migrant related initiatives, the reduced communication and participation due to limited language proficiency, and the challenges of engaging migrants as priorities to address for responsive health system policy development (see Figure 3). For challenges with implementing responsive health system policies, the top ranked challenge was policy not aligning with decision-makers’ objectives (see Figure 4). 

**Phase 3**: The results for the final phase are presented in Appendix Table A4 and the response rate for this phase was 76%. The majority of stakeholders supported the terms used to describe the disadvantaged migrants. The results from this survey of the Delphi process achieved 100% consensus on issues around scoping migrant related initiatives and 77% consensus on data identification and analyses. 

### 3.3. Rank Shift Analysis and Non-Responders

Rank shift analysis on building a responsive health system and engaging migrant communities indicated a substantial *shift* in agreement among experts from Survey 2 to the final survey. Survey 3 found a 36% shift in agreement among stakeholder on ranking *health inequities* first. For challenges in engaging migrant communities, 25% shift in agreement was computed on ranking “unfamiliarity with interpreters, translators and cultural brokers as the highest challenge”. Descriptive statistics comparing the two non-responders to the stakeholders showed no significant difference on gender and country of current practice.

## 4. Discussion

As worldwide migration increases, the media, decision makers and stakeholders are recognizing the importance of building responsive health and other social systems. This Delphi consensus highlights key policy priorities in this process including raising awareness of challenges facing decision makers, monitoring health inequities, responsiveness for time pressures and limited local language proficiency. If policies and programs for a responsive health system can be developed, crises may be averted in the future [14].

Similar to the literature, disadvantaged persons affected by mass migration were described as subjected to political and health system exclusion, subgroups of migrant populations at risk of health inequities, and local populations that face competition from newly arriving migrant populations. Additional factors include risk of isolation, discrimination, deterioration in health, and economic productivity [15]. 

Participants identified International Organization of Migration Health Equity Impact Assessment (HEIA) [16], WHO essential drug program, evidence based migrant health guidelines, and community mediators as the top resources on health systems for disadvantaged populations. Existing population health research does not provide subgroup analyses and often does not provide decision makers with clear evidence on unique health system elements that would be relevant for migrants. The Migrant Integration Policy Index (MIPEX) tool is beginning to address this gap. This tool measures policies to integrate migrants in all EU migrant Member states, as well as evaluating and comparing what countries are doing to promote the integration and health care of migrants. (MIPEX http://www.mipex.eu/health).

Pressured policy process was ranked as one of the most important health system issues to address. WHO and other organizations have used this point to push for responsive health systems before a crisis begins to mitigate population harms (WHO as above in intro). Rapid changes in the profile of migrants, politics, and limited resources challenge health system responsiveness. Without foresight, both low income migrants and non-migrants living in the receiving communities may suffer from inadequate systems. Integrating the HEIA tool within health system policy planning could support more equitable health service provision and strengthen the overall healthcare system [15,17,18,19]. 

Health equity was identified as a policy priority for responsive health systems [15,19]. Often, health needs of migrants are poorly understood, communication between health care providers and migrant clients remains poor, and health systems are not prepared to respond adequately [20]. High quality data on health determinants, health status and health service utilization by migrants are beginning to emerge from large linked database studies [21]. Scoping, data identification, and data analysis is vital for developing effective health system policy. Data are also needed to prevent unintended harms [15] and to judge the benefits outweigh harms of new policies.

Engaging migrant communities was identified as a key policy challenge. To ensure the engagement of migrants and other disadvantaged communities, countries need to support provision of culturally sensitive health systems to help empower migrants that in turn will increase their involvement. Further, governments need to prioritize data collection on disadvantaged communities, including migrants, to ensure a more holistic understanding of health.

### 4.1. Strengths and Limitations 

The international interdisciplinary stakeholder from 13 countries and showed a good response rate and completion rate (≥80%). This study has contributed to building shared vocabulary and processes that can be used at international migrant policy meetings and subsequent implementation of emerging recommendations. 

Overall, there was not a high number of participants. Notably, there was a limited number of experts from Africa, South East Asia, and South Asia. Participants did not include recent refugees, other migrants and people affected by migration. The Delphi required experts to communicate in English. A few experts found the wording/nomenclature of policy process questions complicated and difficult to understand. 

### 4.2. Implications for Policy and Research 

Studies from across the EU demonstrate considerable, but varied, health inequities between migrants and non-migrants [21]. There is growing recognition that migrants face unique obstacles in accessing health services, such as lack of information, cultural and linguistic barriers, and socioeconomic deprivation. Therefore, effective migrant integration policies need to be developed that facilitate access to health services. Providing universal healthcare coverage could also be more cost effective than providing emergency health care, justifying the expansion and improvement of healthcare services for migrants. A recent study in Germany demonstrated for example that if all asylum seekers had the same access to the healthcare system, total spending for medical care over the past 20 years could have been cut by 22% [22]. The emergence of systematic reviews in the field of migrant health [22] will play an important role in supporting policy development as it has in other health system areas [23].

Migrant have overburdened the health systems of Europe, causing fundamental deficiencies in the infrastructure, guidelines, referral mechanisms, responsive funding and resources, preparedness and staff support [24]. Conditions of vulnerability have also increased due to the length of stay of migrants at transit and reception centers, with a worsening in their physical and mental health. Anti-migrants sentiment, xenophobia and populism have determined more restrictive migration policies and cut of funding and support towards integration efforts. 

## 5. Conclusions 

The results of this Delphi consensus highlight some of the unique heath system needs that result from migration. Specifically, the processes identified aim to explicitly and systematically incorporate migrant needs into responsive health systems, to avoid crisis and ensure non-discrimination and equal entitlement to health services. Leading policy initiatives included the Health Equity Impact Assessment, WHO Essential Drug List, evidence based migrant health guidelines and the International Organization for Migration annual reports. All are equipped to help address inequities, racism and discrimination as well as addressing unique clinical needs. Responsive health systems need migrant engagement and migrant relevant program and policy development. 

## Figures and Tables

**Table 1:** Demographic and characteristics of panel experts.

Variable	n	(%)
Age (years)		
≤30	2	(5.6)
31–40	6	(16.7)
41–50	10	(27.8)
51–60	12	(33.3)
>60	6	(16.7)
Sex		
Male	20	(48.8)
Female	21	(51.2)
Country of current practice		
Australia	6	(14.6)
Belgium	1	(2.4)
Canada	9	(22.0)
China	1	(2.4)
Denmark	1	(2.4)
Germany	1	(2.4)
Greece	1	(2.4)
Italy	1	(2.4)
Lebanon	3	(7.3)
Malaysia	1	(2.4)
The Netherlands	4	(9.8)
Spain	1	(2.4)
Sweden	3	(7.3)
United States of America	8	(19.5)
Mother tongue		
English	17	(47.2)
Other	19	(52.7)
Current professional role		
Primary health care practitioner	11	(26.8)
Public health/surveillance professional	6	(14.6)
Migration policy developer	2	(4.8)
Migration health researcher	14	(34.2)
Health impact assessment developer	1	(2.4)
Other	6	(14.6)
Length of time researching/practicing/working with migrants		
≤5 years	5	(15.6)
6–10 years	11	(34.4)
11–15 years	6	(18.6)
>16 years	10	(31.3)
Length of time in migrant research/policy		
≤5 years	11	(34.4)
6–10 years	10	(31.3)
11–15 years	6	(18.8)
>16 years	5	(15.6)

## Appendix A

**Table A1:** Describing Disadvantage Persons Affected by Migration.

What Are the Populations of Interest in Order to Develop Responsive, Socially Cohesive and Responsive Health Systems in a Time of Migration?			
Subgroups of Migrants (at Risk for Health Inequities)	The “Excluded” (Political and System Exclusion)	Competition from Other Non-Migrant Populations	Outlier: Intersectionality (Piecing It All Together)
Undocumented families Rural workers New migrants in urban slums Unaccompanied minors Migrant with low literacy *[Migrant]* pregnant women People in detention centers Forced migrants, asylum seekers Trafficked women Members of sexual minorities Those subject to xenophobia Survivors of torture Low wage migrants in Arab countries New Spanish speaking rural migrant workers Vulnerable Refugees, immigrants and temporary foreign workers with specific needs	The broken is systematic and does not lie in the population itself Rather sets of social, political, and discriminatory and generation factors, place migrants at risk Vulnerabilities often due to structural marginalization Issues generating a gap between this population pocket and the social and healthcare services Discrimination with respect to access to employment, low wages, and additional barriers for immigrant women	Refugees receive support while other disadvantaged communities don’t—primary and secondary populations Disadvantaged populations may include migrants or receiving country communities Communities receiving the migrants share the burden The secondary population represents vulnerable individuals living within a jurisdiction, whose health and wellbeing is affected by patterns of migration in their community	I prefer to speak of diversity where possible, and this emphasizes the status between immigrant status, ethnicity, and inequity (an intersectional approach).

**Table A2:** Percentage of Stakeholder Usage of Practice Based Resources and Tools for Migration.

Tools/Resources	(%) of Experts Who Have Used the Tools/Resources	
	n	(%)
1. International Organization for Migration Publications (*n* = 30)	18	(60.0)
2. Community Engagement Guidelines (*n* = 26)	15	(57.7)
3. Migrant Health Expert Opinion guidelines (*n* = 36)	15	(53.6)
4. Community Health Mediators (*n* = 28)	13	(46.4)
5. WHO Essential Drug Program (*n* = 28)	13	(44.8)
6. Evidence Based Migrant Health Guidelines (*n* = 28)	9	(32.2)
7. Health (Equity) Impact Assessment (*n* = 27)	9	(33.3)
8. Machine translation for medical care (*n* = 28)	5	(17.9)
9. Migration Integration Policy Index (MIPEX) (*n* = 26)	3	(11.5)

**Table A3:** Percentage of Experts Aware of Practice Based Resources and Migration.

Tools/Resources	(%) of Experts Aware of the Tools/Resources	
	n	(%)
1. International Organization for Migration Publications (*n* = 30)	26	(86.7)
2. Health (Equity) Impact Assessment (*n* = 29)	22	(79.3)
3. WHO Essential Drug Program (*n* = 28)	21	(75.0)
4. Evidence Based Migrant Health Guidelines (*n* = 29)	19	(69.0)
5. Community Health Mediators (*n* = 29)	18	(62.1)
6. Migrant Health Expert Opinion guidelines (*n* = 30)	18	(60.0)
7. Machine translation for medical care (*n* = 29)	15	(51.7)
8. Community Engagement Guidelines (*n* = 29)	15	(51.7)
9. Migration Integration Policy Index (MIPEX) (*n* = 30)	11	(36.7)

**Table A4:** Percentage of Experts’ Agreement on Policy Priorities.

The Main Concerns	% of Experts Agreement ^1^
(*n* = 32)	(%)
Describing disadvantaged populations	(83.9)
Building a responsive health system	(87.1)
Defining “*scope of migrant related initiatives*”	(100.0)
Data identification and analysis	(77.4)
Engaging migrant communities	(83.9)
Implementing evidence-informed policy	(89.6)

^1^ Percentage of experts supporting the ranking of *challenges* identified for each concern in phase 2.
